# A hybrid protein is a functional molecule to reduce the cytokine storm caused by excessively activated macrophages

**DOI:** 10.1111/imcb.70000

**Published:** 2025-02-15

**Authors:** Masaki Ikemoto, Takuya Kotani, Kohki Okada, Shogo Matsuda, Tohru Takeuchi

**Affiliations:** ^1^ Division of Rheumatology, Department of Internal Medicine IV Osaka Medical and Pharmaceutical University Osaka Japan; ^2^ Department of Medical Technology and Sciences, Faculty of Health Sciences Kyoto Tachibana University Kyoto Japan

**Keywords:** Human S100 proteins, hybrid protein, inflammatory cytokines, macrophages, NF‐κB

## Abstract

We recently developed a hybrid protein, tentatively named human MIKO‐1 (hMIKO‐1), based on the amino acid sequences of human S100A8 (hS100A8) and hS100A9. Human THP‐1 macrophages (THP‐1m), differentiated from THP‐1 cells by phorbol 12‐myristate 13‐acetate, were used to investigate the immune function of hMIKO‐1 as a drug for inflammatory diseases. Western blotting was conducted to confirm whether hMIKO‐1 binds with β‐actin and nuclear factor‐kappa B to form complexes in THP‐1m. A polymerase chain reaction (PCR) and quantitative PCR were performed to examine changes in the messenger RNA levels of proinflammatory cytokines in THP‐1m. Fluorescent immunochemical staining was used to observe the intracellular localization of hMIKO‐1 and hS100A8 or hS100A9 in THP‐1m. As observed microscopically, the intracellular localization of hMIKO‐1 in THP‐1m was consistent with that of hS100A8, suggesting the close involvement of hS100A8 in the intracellular behavior of hMIKO‐1 in THP‐1m. Western blotting revealed that hMIKO‐1 formed complexes with intracellular proteins, such as β‐actin and nuclear factor‐kappa B, to negatively regulate inflammatory signal transduction in THP‐1m. Flow cytometry showed that the binding of hMIKO‐1 to THP‐1m significantly decreased when THP‐1m were preliminarily treated with a sialidase (neuraminidases) cocktail. Therefore, the present results strongly suggest that the binding of hMIKO‐1 to THP‐1m closely involves the sugar chains of the surface proteins of cells. The messenger RNA expression of each proinflammatory cytokine was significantly suppressed in THP‐1m preliminarily treated with hMIKO‐1 despite a subsequent stimulation with lipopolysaccharide. In conclusion, hMIKO‐1 is a functional molecule that significantly inhibits inflammatory signal transduction in THP‐1m.

## Graphical abstract

In this article, we have developed a hybrid protein, tentatively named human MIKO‐1 (hMIKO‐1). The messenger RNA expression of each proinflammatory cytokine was significantly suppressed by hMIKO‐1 despite a subsequent stimulation with lipopolysaccharide. hMIKO‐1 is a functional molecule that significantly attenuates the cytokine storm.

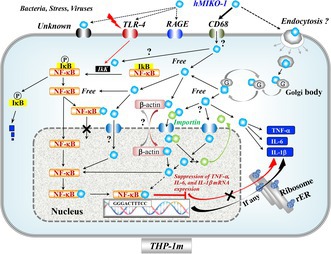

## INTRODUCTION

Human S100A8 (hS100A8) and hS100A9 (hS100 proteins) are proteins with various immune functions in inflammatory diseases, such as rheumatoid arthritis,^1^, ^2^ inflammatory bowel diseases,^3^, ^4^, ^5^ including ulcerative colitis,^6^ Crohn's disease^7^ and acute hepatitis.^8^, ^9^ hS100 proteins and their heterodimers (hS100A8/A9 or calprotectin) are derived from immune cells of myeloid origin, such as monocytes/macrophages and neutrophils. These proteins are closely involved in acute inflammation.^10^, ^11^, ^12^, ^13^, ^14^, ^15^ In inflamed tissues, large amounts of hS100 proteins and calprotectin are secreted from immune cells in response to inflammatory events in the body, with calprotectin reportedly exhibiting various immune functions in a concentration‐dependent manner.^16^ Macrophages may be constantly exposed to many antigenic substances, for example, microorganisms and their components, in the body, and these cells are generally activated via various receptors, such as Toll‐like receptors (TLRs)^17^, ^18^, ^19^ and the receptor for advanced glycation end products (RAGE).^20^, ^21^, ^22^ In acute inflammation, macrophages actively secrete proinflammatory cytokines, including tumor necrosis factor‐alpha, interleukin‐6 and interleukin‐1β, to defend the whole body from attacks by these antigenic substances. hS100 proteins reportedly cooperate to maintain internal homeostasis in the body by negatively regulating the excessive activation of macrophages.^11^, ^12^, ^13^, ^14^ Therefore, hS100 proteins are important target molecules in our strategy to develop new drugs aimed at treating the aforementioned inflammatory diseases.

**Figure 1:**
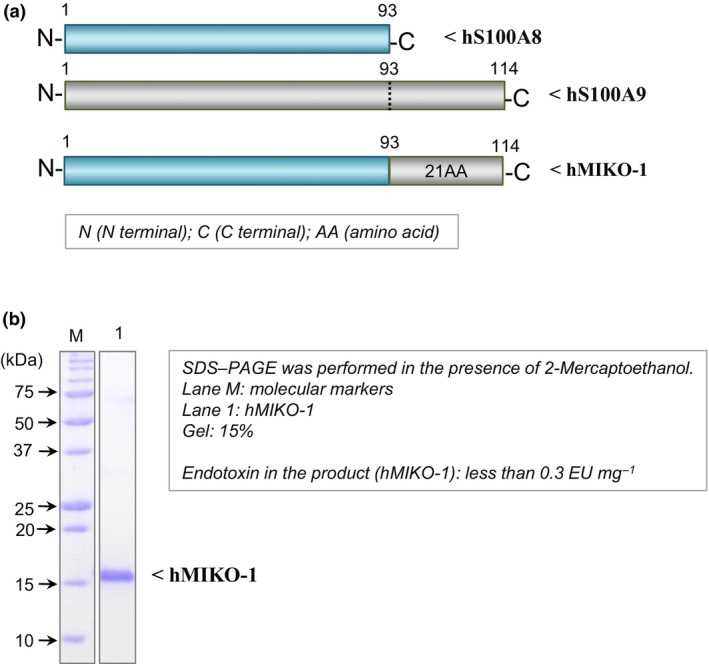
Design of a hybrid protein (hMIKO‐1) and its purification. **(a)** The frames of the full‐length AA sequences of hS100A8 and hS100A9 were schematically drawn by blue and gray horizontal bars, respectively. hMIKO‐1 is a hybrid protein of hS100A8 and hS100A9. The whole frame of hMIKO‐1 was as follows: 21 AA residues from the C terminus of hS100A9 were added to the C terminus of hS100A8. **(b)** SDS–PAGE (15% gel) was performed to confirm the purity of hMIKO‐1 in the absence of 2‐mercaptoethanol. hMIKO‐1 was purified by affinity chromatography (Ni‐agarose; Nacalai Tesque, Kyoto, Japan), ion‐exchange chromatography (DEAE cellulose; Bio‐Rad Co., Ltd, USA) and gel filtration (Sephacryl S‐300 HR). Proteins in the gel were stained with Coomassie Brilliant Blue. Lanes M and 1 were molecular markers and purified hMIKO‐1, respectively. As shown in the kit (Toxinsensor, Endotoxin Detection System; GenScript Inc., USA), the concentration of contaminated endotoxin present in the product hMIKO‐1 was less than 0.3 EU mg^−1^ protein. This result indicates no significant effects in subsequent experiments (data not shown). AA, amino acid; PAGE, polyacrylamide gel electrophoresis; SDS, sodium dodecyl sulfate.

The functional domain of proteins may be biochemically concealed in their amino acid (AA) sequences or three‐dimensional structures. Despite extensive efforts, the domain of each hS100 protein that is responsible for its activity has yet to be identified. We recently indicated that the N‐ and C‐terminal regions of rat S100A8 (rS100A8) and rS100A9, respectively, are important key regions for the immune function of rat macrophages.^23^ Hybridization is a simple and effective strategy for discovering new functional molecules or useful therapeutic drugs.^24^ In our pioneering studies, we developed a hybrid protein, rat MIKO‐1 (rMIKO‐1), and reported its potential as a novel regulator of experimental colitis in rats.^25^ Based on our previous findings, we hypothesized that a human hybrid protein (tentatively named hMIKO‐1) is capable of reducing the excessive activation of macrophages by inhibiting the messenger RNA (mRNA) expression of proinflammatory cytokines. To prove our hypothesis, we designed hMIKO‐1 based on the AA sequences of hS100A8 and hS100A9 and prepared hMIKO‐1 as previously described (Figure 1a).^25^ To clarify the properties of hMIKO‐1, cells (THP‐1m) differentiated from THP‐1 cells, by phorbol 12‐myristate 13‐acetate (PMA), from 4 to 5 days were used in this study.

We herein investigated whether hMIKO‐1 is a functional molecule that negatively regulates inflammatory reactions and, thus, has potential in the treatment of intractable diseases, including interstitial pneumonia and scleroderma, with systemic inflammatory reactions.

## RESULTS

### Expression and purification of hMIKO‐1

After sodium dodecyl sulfate–polyacrylamide gel electrophoresis (SDS–PAGE), proteins in the gel were stained with Coomassie Brilliant Blue. hMIKO‐1 was successfully purified as a monomeric protein with high purity in SDS–PAGE (Figure 1b). Contaminated endotoxin was deleted as much as possible using a DEAE cellulose (Macro‐Prep High Q Media, Bio‐Rad, USA) column [26 mm (D) × 80 mm (L)]. As measured using a kit (Toxinsensor, Endotoxin Detection System; GenScript Inc., USA), the concentration of endotoxin in samples was < 0.3 EU mg^−1^ protein, indicating almost no effects in subsequent experiments (data not shown).

### Differentiation of THP‐1 to THP‐1m by PMA

**Figure 2:**
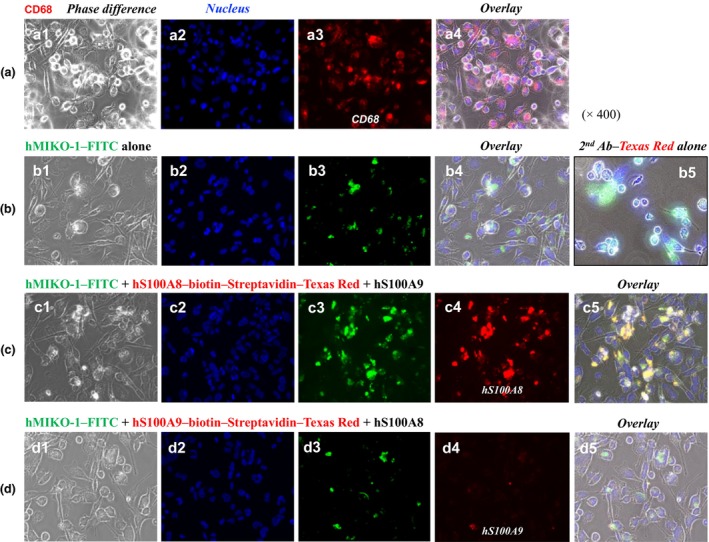
The close involvement of hS100A8 and hS100A9 in the uptake of hMIKO‐1 into THP‐1m. FICS was performed to clarify the close involvement of hS100A8 and hS100A9 in the uptake of hMIKO‐1 into THP‐1m using hS100A8, hS100A8–biotin, hS100A9, hS100A9–biotin and streptavidin–Texas Red and a specific antibody for CD68. **(a)** CD68 was stained using an anti‐CD68 antibody and anti‐mouse IgG (horse) IgG–Texas Red conjugate. **(b)** THP‐1m cells were treated with hMIKO‐1–FITC alone. An anti‐mouse IgG (horse) IgG (secondary antibody)–Texas Red conjugate alone was used to examine its nonspecific binding to cells **(b5)**. **(c)** THP‐1m cells were treated with a mixture of hMIKO‐1–FITC, hS100A8–biotin and hS100A9, in which hS100A8–biotin was detected with the Streptavidin–Texas Red conjugate. **(d)** THP‐1m cells were treated with a mixture of hMIKO‐1 cells FITC, hS100A8 and hS100A9–biotin, in which hS100A9–biotin was detected with the streptavidin–Texas Red conjugate. **(a1, b1, c1, d1)** Phase difference, **(a2, b2, c2, d2)** nucleus, **(a3)** (CD68), **(b3, c3, d3)** hMIKO‐1, **(c4)** hS100A8, **(d4)** hS100A9 and **(a4, b4, c5, d5)** overlay. hMIKO‐1–FITC (approximately 5 × 10^−2^ g L^−1^ each), hS100A8 (approximately 3.3 × 10^−2^ g L^−1^) and hS100A9 (approximately 5 × 10^−2^ g L^−1^) were used. Microscopic magnification (× 400). The nucleus of THP‐1m was stained with DAPI in the VECTASHIELD mounting medium. Microscopic images were observed using fluorescent microscopy (BIOREVO BZ‐9000; KEYENCE Co., Ltd, Osaka). Ab, antibody; DAPI, 4′,6‐diamidino‐2‐phenylindole dihydrochloride; FICS, fluorescent immunochemical staining; FITC, fluorescent 5‐isothiocyanate; hMIKO‐1, human MIKO‐1; Ig, immunoglobulin.

THP‐1 cells were incubated with PMA (100 nmol L^−1^ as the final concentration) at 37°C in a 5% CO_2_ incubator for 4–5 days. As observed microscopically, THP‐1 cells, but not all cells, morphologically differentiated to THP‐1m. Immunochemically, THP‐1m cells were identified using a specific antibody (KP1; Exalpha Biologicals Inc., USA) for CD68 on the membranes of cells by fluorescent immunochemical staining (FICS; Figure 2a).

### Dynamic mobility of hMIKO‐1 in THP‐1m

#### Immunological roles of hS100A8, hS100A9 and calprotectin (hS100A8/A9) in the uptake of hMIKO‐1 into THP‐1m

When THP‐1m cells were treated with hMIKO‐1–fluorescent 5‐isothiocyanate (FITC) isomer alone for 12 h, hMIKO‐1–FITC bound to these cells (Figure 2b, panel b3). More hMIKO‐1–FITC bound to THP‐1m treated with a mixture of hMIKO‐1–FITC, hS100A8–biotin and hS100A9 than to those treated with hMIKO‐1–FITC alone (Figure 2c, panel c3). Markedly less hS100A9‐biotin bound to THP‐1m treated with a mixture of hMIKO‐1–FITC, hS100A8 and hS100A9–biotin than to those treated with hS100A8–biotin alone (Figure 2d, panel d4). These results suggest the important role of hS100A8 in the binding of hMIKO‐1–FITC to THP‐1m, leading to its uptake into these cells. The nonspecific binding of an anti‐mouse immunoglobulin G (IgG; horse) IgG–Texas Red (TR) conjugate was not observed (Figure 2b, panel b5).

#### Intracellular localization of hMIKO‐1 and β‐actin

**Figure 3:**
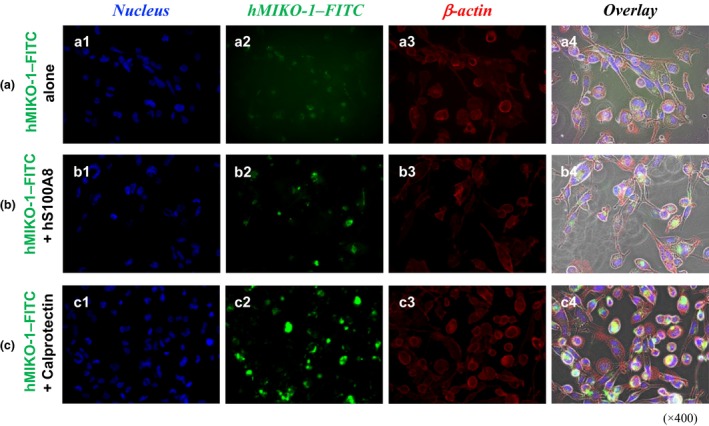
Potential activity of calprotectin involving the uptake of hMIKO‐1 in THP‐1m. **(a)** THP‐1m cells were treated with hMIKO‐1–FITC alone. **(b)** THP‐1m cells were treated with a mixture of hMIKO‐1–FITC and hS100A8. **(c)** THP‐1m cells were treated with a mixture of hMIKO‐1–FITC and calprotectin. **(a1–c1)** nucleus, **(a2–c2)** (hMIKO‐1), **(a3–c3)** β‐actin and **(a4–c4)** overlay. hMIKO‐1–FITC (approximately 5 × 10^−2^ g L^−1^ each), hS100A8 (approximately 3.3 × 10^−2^ g L^−1^), hS100A9 (approximately 5 × 10^−2^ g L^−1^) and calprotectin (approximately 5 × 10^−2^g L^−1^) were used. Microscopic magnification ×400. The nuclei of THP‐1m cells were stained with DAPI in the VECTASHIELD mounting medium. β‐Actin in THP‐1m was stained using an anti‐β‐actin monoclonal antibody (approximately 2 × 10^−3^ g L^−1^) and anti‐mouse IgG (horse) IgG–Texas Red conjugate (approximately 2 × 10^−3^ g L^−1^). All microscopic images were observed using fluorescent microscopy (BIOREVO BZ‐9000, KEYENCE Co., Ltd, Osaka). Microscopic magnification (× 400). DAPI, 4′,6‐diamidino‐2‐phenylindole dihydrochloride; FITC, fluorescent 5‐isothiocyanate; hMIKO‐1, human MIKO‐1; Ig, immunoglobulin.

FICS was performed to visually observe the intracellular localization of hMIKO‐1 and β‐actin in THP‐1m using a fluorescent microscope. The intracellular localization of hMIKO‐1–FITC in THP‐1m was similar to that of β‐actin (Figure 3c, panels c2, c3). Microscopically, more hMIKO‐1–FITC bound to THP‐1m treated with the mixture of hMIKO‐1–FITC and calprotectin (Figure 3a, c). Although microscopic images of hMIKO‐1–FITC taken into THP‐1m were unclear (Figure 3c, panel c2), hMIKO‐1–FITC appeared to colocalize with β‐actin in these cells (Figure 3c, panel c4).

#### Estimation of the receptor‐like molecule against hMIKO‐1 on THP‐1m

**Figure 4:**
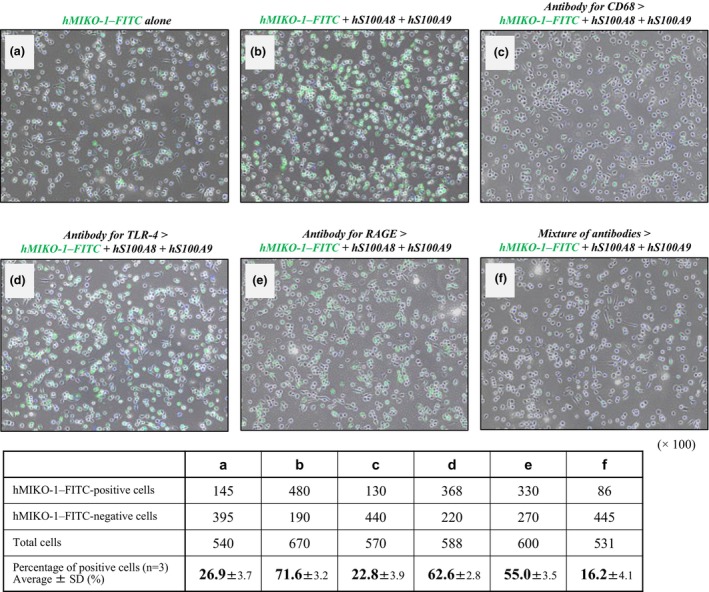
Inhibitory assay of the binding of hMIKO‐1 to THP‐1m and its uptake in cells. FICS was performed to identify specific receptor‐like molecules on THP‐1m for hMIKO‐1 using specific antibodies for CD68, TLR4, RAGE and their mixture. **(a)** THP‐1m cells were treated with hMIKO‐1–FITC alone at 37°C for 3 h in 5% CO_2_. **(b)** THP‐1m cells were treated with a mixture of hMIKO‐1–FITC, hS100A8 and hS100A9. **(c–f)** THP‐1m cells were preliminarily treated with each antibody (approximately 1 × 10^−2^ g L^−1^) specific for CD68, TLR4, RAGE and their mixture, respectively, at 37°C for 1 h in 5% CO_2_. After washing, THP‐1m were treated with a mixture of hMIKO‐1–FITC (approximately 5 × 10^−2^ g L^−1^), hS100A8 (approximately 3.3 × 10^−2^ g L^−1^) and hS100A9 (approximately 5 × 10^−2^ g L^−1^) for 3 h. hMIKO‐1–FITC cells were then microscopically observed using fluorescent microscopy (BIOREVO BZ‐9000, KEYENCE Co., Ltd, Osaka). Positive cells were counted by a cell counter. The results obtained are summarized in the table at the bottom of the figure. Microscopic magnification (× 200). FICS, fluorescent immunochemical staining; FITC, fluorescent 5‐isothiocyanate; hMIKO‐1, human MIKO‐1; RAGE, receptor for advanced glycation end products; TLR, Toll‐like receptor.

FICS was performed to microscopically confirm the contribution of CD68, TLR4 and RAGE to the uptake of hMIKO‐1–FITC into THP‐1m. In THP‐1m treated with hMIKO‐1–FITC in the presence of hS100A8 and hS100A9, the binding of hMIKO‐1–FITC to cells markedly increased (Figure 4b); however, under the same conditions, this binding was strongly inhibited by the anti‐human CD68 monoclonal antibody KP1 (1 × 10^‐2^  g L^−1^; Figure 4c). By contrast, in THP‐1m treated with a specific antibody for TLR4 or RAGE, the binding of hMIKO‐1–FITC to THP‐1m was less than that in cells treated with CD68 (Figure 4d, e). The binding of hMIKO‐1–FITC to THP‐1m was strongly blocked by a mixture of the three antibodies (Figure 4f). These results suggest the close involvement of CD68 in the binding of hMIKO‐1–FITC to THP‐1m and its uptake inside cells, with CD68 being one of the important receptor‐like proteins on the membrane of THP‐1m.

### Effects of sugar chains of CD68 on the dynamic mobility of hMIKO‐1–FITC in THP‐1m

#### Analysis by flow cytometry

**Figure 5:**
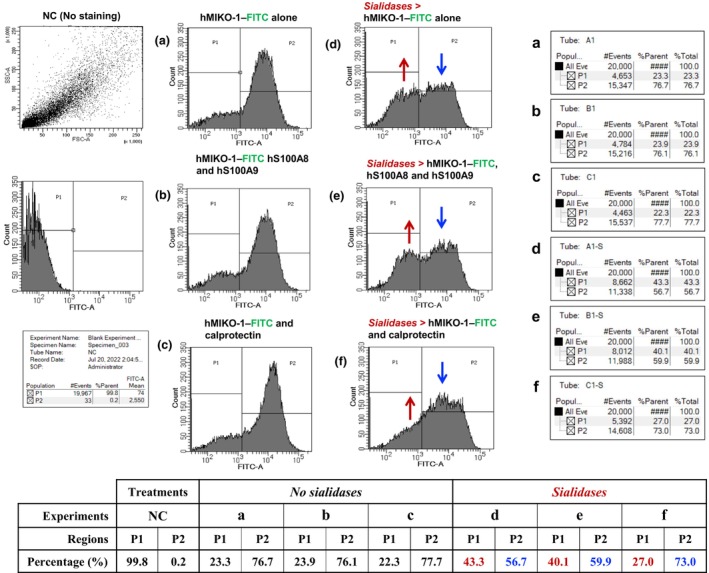
Functional role(s) of sugar chains of CD68 on THP‐1m. Flow cytometry was performed to confirm whether the sugar chains of CD68 on THP‐1m cells were involved in the binding of hMIKO‐1–FITC to THP‐1m. In the present study, THP‐1m cells (approximately 5 × 10^5^ mL^−1^), which were resuspended in an adequate volume of buffer B, were treated with **(d–f)** or without **(a–c)** sialidases/neuraminidases at room temperature for 30 min according to the manufacturer's instructions. THP‐1m cells were treated with hMIKO‐1–FITC alone **(a, d)**, hMIKO‐1–FITC + hS100A8 + hS100A9 **(b, e)**, or hMIKO‐1–FITC + calprotectin **(c, f)**. The final concentrations of hMIKO‐1–FITC, hS100A8, hS100A9 and calprotectin were approximately 5, 3.3, 5 and 5 × 10^−2^ g L^−1^, respectively. After washing with buffer B, cells were resuspended in an adequate volume of buffer B and subjected to flow cytometry. The two groups (P1 and P2) are shown in the histogram of negative control. The percentages of the P1 and P2 regions against all events are summarized in the table at the bottom of the figure. This experiment was repeated three times (*n* = 3). As we obtained similar results, we showed representative data in the present study. FITC, fluorescent 5‐isothiocyanate; hMIKO‐1, human MIKO‐1; NC, negative control.

To clarify the immunological significance of sugar chains composed of CD68 on THP‐1m, flow cytometry was performed based on a previously reported method.^23^ The grouping of THP‐1m was not performed in a dot blot analysis because only THP‐1m cells were used. The two groups (P1 and P2) are shown in the histogram of the negative control (Figure 5). Upon the treatment of THP‐1m with hMIKO‐1–FITC (approximately 5 × 10^‐3^ g L^−1^) alone, hMIKO‐1–FITC bound to most THP‐1m, while the weaker binding of hMIKO‐1–FITC to THP‐1m was also noted (Figure 5a). As described above, more hMIKO‐1–FITC bound to THP‐1m in the presence of hS100A8 and hS100A9 (Figure 5b) or calprotectin (Figure 5c). These results suggest that hS100A8 and hS100A9 or calprotectin are closely involved in the binding of hMIKO‐1–FITC to THP‐1m. In THP‐1m treated with a sialidase (neuraminidases) cocktail, the number of cells bound to hMIKO‐1–FITC significantly decreased, as indicated by blue vertical arrows (P2). By contrast, the number of THP‐1m cells that weakly bound to hMIKO‐1–FITC (P1) increased, as indicated by red vertical arrows (Figure 5d–f). Therefore, the sugar chains of some surface proteins, such as CD68 and other receptor‐like proteins, on the membrane of THP‐1m appear to be important elements in the binding of hMIKO‐1–FITC to or its uptake into these cells.

#### Western blotting after immunoprecipitation

**Figure 6:**
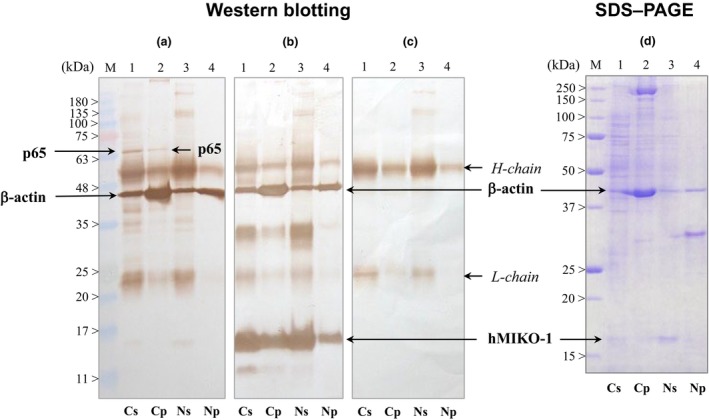
Identification of intracellular molecules binding with hMIKO‐1. Western blotting and SDS–PAGE were conducted to identify intracellular molecules binding with hMIKO‐1. The extraction of proteins in the cytoplasm and nucleus of THP‐1m was performed using the nuclear/Cytosol Fractionation Kit (K266‐25) according to the manufacturer's instructions. Immunoprecipitation was performed using cytoplasmic and nuclear protein samples extracted from the cytoplasm and nucleus of THP‐1m and two specific monoclonal antibodies (mAbC5 and mAbC6) for hMIKO‐1, in which protein samples were treated with hMIKO‐1 (approximately 2 × 10^−3^ g L^−1^) with gentle mixing at 4°C for 12 h. After centrifugation, supernatants (Cs and Ns) and pellets (Cp and Np) were obtained and subjected to Western blotting. Western blotting was performed to confirm p65, β‐actin and hMIKO‐1. **(a)** hMIKO‐1 and β‐actin, (**b**) hMIKO‐1, β‐actin and the mouse IgG (heavy and light chains), **(c)** heavy and light chains of the mouse IgG in each sample. **(a)** p65 and β‐actin were stained using anti‐p65 and β‐actin monoclonal antibodies as the first antibody and anti‐mouse IgG (horse) IgG–HRP conjugates as the second antibody. **(b)** hMIKO‐1 and β‐actin were stained using specific monoclonal antibodies for hMIKO‐1 (mAbC5) and β‐actin, respectively, as the first antibody and an anti‐mouse IgG (horse) IgG–HRP conjugate was used as the second antibody. **(c)** Mouse monoclonal IgG was stained using an anti‐mouse IgG (horse) IgG–HRP conjugate. SDS–PAGE (15% gel) was performed in the presence of 2‐ME to visually show coprecipitated proteins with hMIKO‐1 in the cytoplasm and nucleus of THP‐1m. **(d)** Protein bands were stained with Coomassie Brilliant Blue. Lane M: Molecular markers. Lanes 1 (Cs) and 3 (Ns): Proteins in the supernatant after immunoprecipitation. Lanes 2 (Cp) and 4 (Np): Coprecipitated proteins in the pellet after immunoprecipitation. C and N indicate the cytoplasm and nucleus, respectively. 2‐ME, 2‐mercaptoethanol; HRP, horseradish peroxidase; hMIKO‐1, human MIKO‐1; Ig, immunoglobulin; PAGE, polyacrylamide gel electrophoresis; SDS, sodium dodecyl sulfate.

In this experiment, we found that hMIKO‐1 formed complexes with some intracellular constituents in THP‐1m cells. The results obtained showed a clear protein band of β‐actin in each precipitate derived from the cytoplasm and nucleus of THP‐1m (Figure 6a). In addition, a slightly positive band of p65 was detected only in the precipitate derived from the cytoplasmic fraction (Figure 6a, lanes 1 and 2). hMIKO‐1 was detected in both the cytoplasm and nucleus of THP‐1m (Figure 6b, lanes 1–4). This result strongly suggests that hMIKO‐1 is taken into the cytoplasm of THP‐1m and further migrates inside the nucleus. Additionally, heavy and light chains of the first antibody are indicated as the control (Figure 6c, lanes 1–4). The protein bands present in the supernatant and precipitate were stained with Coomassie Brilliant Blue, as shown in Figure 6d.

#### Inhibitory effects of hMIKO‐1 on the mRNA expression of proinflammatory cytokines in THP‐1m

**Figure 7:**
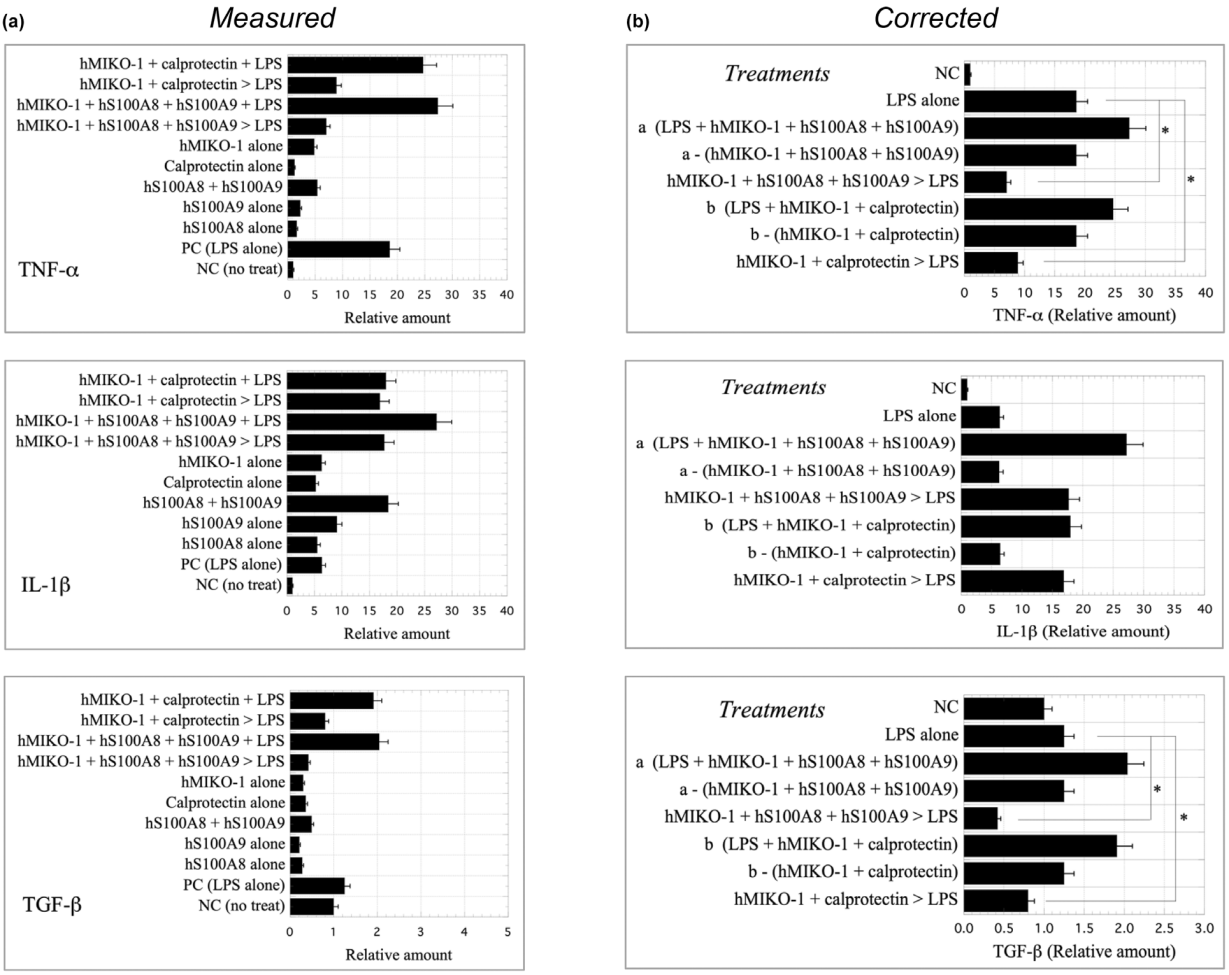
Inhibitory effects of hMIKO‐1 on the mRNA expression of three types of cytokines (TNF‐α, IL‐1β and transforming growth factor beta) in THP‐1m. To investigate the inhibitory effects of hMIKO‐1 on the mRNA expression of TNF‐α, IL‐1β and transforming growth factor beta, the mRNAs of these cytokines were quantified using a StepOnePlus Real‐Time PCR System (Applied Biosystems, Foster City, CA 94404, USA). Values are indicated by horizontal bars, in which the value for each cytokine was calculated as the average of the data obtained (*n* = 5). Assay conditions are indicated on the left side of each graph. **(a)** All measured values under the condition indicated are shown by horizontal bars. Briefly, “hMIKO‐1 + calprotectin + LPS” were treated with the mixture of hMIKO‐1, calprotectin and LPS for 1 h. “hMIKO‐1 + calprotectin > LPS”: treated with LPS alone for 1 h after the treatment with the mixture of hMIKO‐1 and calprotectin overnight. “hMIKO‐1 + hS100A8 + hS100A9 + LPS”: treated with the mixture of hMIKO‐1, hS100A8, hS100A9 and LPS for 1 h. “hMIKO‐1 + hS100A8 + hS100A9 > LPS”: treated with LPS after the treatment with the mixture of hMIKO‐1, hS100A8 and hS100A9 overnight. “hS100A8 + hS100A9”: treated with the mixture of hS100A8 and hS100A9 overnight. hMIKO‐1, hS100A8, hS100A9 and calprotectin alone: treated with each protein for 1 h. PC: treated with LPS alone for 1 h. hMIKO−1 (approximately 5 × 10^−2^ g L^−1^), hS100A8 (approximately 3.3 × 10^−2^ g L^−1^), hS100A9 (approximately 5 × 10^−2^ g L^−1^), calprotectin, (approximately 5 × 10^−2^ g L^−1^) and LPS (approximately 2 × 10^−3^ g L^−1^) were used in this study. NC: no treatment. **(b)** Corrected values are indicated by horizontal bars, with the mRNA level obtained by the treatment of hMIKO−1, hS100A8, hS100A9 or calprotectin alone being subtracted from the measured values obtained under each condition described above. *P < 0.01. hMIKO‐1, human MIKO‐1; IL, interleukin; LPS, lipopolysaccharide; mRNA, messenger RNA; PC, positive control; PCR, polymerase chain reaction; TGF‐β, transforming growth factor‐beta; TNF‐α, tumor necrosis factor‐alpha; NC, negative control.

To investigate the antigenicity of hMIKO‐1 for THP‐1m, we examined changes in the mRNA expression of proinflammatory cytokines in cells treated with hMIKO‐1. In THP‐1m treated with hMIKO‐1 (approximately 5 × 10^‐2^ g L^−1^), hS100A8 (approximately 3 × 10^‐2^ g L^−1^), hS100A9 (approximately 5 × 10^‐2^ g L^−1^) or calprotectin (approx. 5 × 10^−2^ g L^−1^) alone, slight increases were observed in the mRNAs of proinflammatory cytokines, such as tumor necrosis factor‐alpha and interleukin‐1β (Figure 7a). These results indicate the weaker antigenicity of each material for THP‐1m. To clarify the true activity of lipopolysaccharide (LPS), we subtracted the antigen‐like activity of hMIKO‐1, hS100A8, hS100A9 and calprotectin alone from the measured values obtained in each trial. The results obtained showed that the mRNA expression of tumor necrosis factor‐alpha and transforming growth factor beta was significantly suppressed in THP‐1m preliminarily treated with the mixture of hMIKO‐1, hS100A8 and hS100A9, or hMIKO‐1 and calprotectin overnight despite the subsequent stimulation with LPS (approximately 2 × 10^−3^ g L^−1^) for 1 h, but not that of interleukin‐1β (Figure 7b). These results suggest that hMIKO‐1 suppressed the mRNA expression of these inflammatory cytokines in THP‐1m; however, the underlying mechanisms have yet to be elucidated.

## DISCUSSION

In this study, we showed the potential of hMIKO‐1 as a novel player that negatively regulates the excessive activation of macrophages by forming complexes with intracellular proteins related to inflammation. We previously demonstrated that rMIKO‐1 markedly attenuated dodecyl sulfate sodium–induced experimental colitis caused by excessively activated macrophages in rats.^25^ This outcome prompted us to develop a new medicine for inflammatory diseases. Hybridization is a simple and effective strategy for discovering new biological agents with higher quality.^24^ We then developed the hybrid protein, hMIKO‐1, and clarified its potential as a novel suppressor of inflammatory reactions.

A previous study reported the close involvement of hS100 proteins in the process of inflammation.^12^ hMIKO‐1 is a hybrid protein of hS100A8 and hS100A9; therefore, it was initially considered to partly inherit the immunological properties of both hS100A8 and hS100A9. However, the immune properties of hMIKO‐1 did not reflect those of hS100A8 and hS100A9. In this study, we found that hMIKO‐1 functioned more effectively as a negative regulator of inflammation. Although the mechanisms underlying the functional role(s) of hMIKO‐1 in THP‐1m have not yet been elucidated in detail, they may be dependent on its three‐dimensional structure, which differs from that of hS100A8 and hS100A9. Thus, hMIKO‐1 may not functionally share the characteristics of both hS100A8 and hS100A9 in THP‐1m. By contrast, it is important to note that the intracellular localization of hMIKO‐1 was consistent with that of β‐actin in THP‐1m. The presence of β‐actin in the cytoplasm and nucleus of THP‐1m is suggestive of the transport of hMIKO‐1 by β‐actin inside the nucleus of THP‐1m (Figure 6). Therefore, hMIKO‐1 may play important role(s) in the nucleus of excessively activated macrophages.

Unexpected infections by microorganisms or viruses in healthy individuals sometimes result in severe inflammatory diseases, including pneumonia in the lungs. In some cases, patients may gradually deteriorate without appropriate treatment and ultimately die. The severity of inflammatory diseases may be mainly attributed to the massive amounts of inflammatory cytokines secreted from excessively activated macrophages in inflamed tissues. Among immune cells, macrophages may quickly move to inflamed tissues in the first stage of inflammation, and secrete oxidizing substances, such as superoxide anions, to protect the whole body from attack by microorganisms, including viruses; however, their excessive secretion may result in tissue damage around inflamed tissues of the body. Accordingly, the most effective approach is to block the first action of macrophages that leads to the secretion of large amounts of inflammatory cytokines. Inhibitors of cyclooxygenase‐2, which produces some prostaglandins, are generally used in the treatment of inflammatory diseases; however, these inhibitors may induce many side effects.^26^, ^27^ Regarding side effects, hMIKO‐1 may have fewer side effects than many medicines because of its focused effects on macrophages via CD68 on their surface membrane.

Upon inflammation, many immune cells in the circulation quickly move to inflamed tissues in the body, in which large amounts of hS100 proteins, including hS100A8/A9 (or calprotectin), are extracellularly secreted from excessively activated immune cells of myeloid origin. We previously reported that the normal serum concentration of hS100A8/A9 in healthy individuals ranged between 3 × 10^−4^ and 5 × 10^−4^ g L^−1^.^28^ Based on the present results, the concentration of calprotectin in inflamed tissues may be hundreds of times higher than that in the peripheral blood of healthy individuals. In these tissues, more proinflammatory cytokines and hS100 proteins, including calprotectin, may be secreted from excessively activated macrophages. The present results showed that more hMIKO‐1 was taken into THP‐1m treated with hS100A8 + hS100A9 (Figure 2c) or calprotectin (Figure 3c). Under these conditions, hMIKO‐1 may show optimal performance in excessively activated macrophages, thereby effectively suppressing inflammatory reactions. As described above, increases in hMIKO‐1 in THP‐1m may suppress the cytokine storm caused by excessively activated THP‐1m. Although the underlying mechanisms currently remain unclear, intracellular hMIKO‐1 may block the activation of inflammatory signal transduction by forming complexes with not only p65 (nuclear factor‐kappa B) (Figure 6), but also hS100A8 and hS100A9 (Supplementary figure 1).^29^, ^30^, ^31^ Therefore, hMIKO‐1 appears to be a multifunctional molecule that forms complexes with limited intracellular proteins to regulate inflammation. This property may be specialized for the immune function of hMIKO‐1 as a novel regulator. However, the mechanisms underlying the molecular behavior of hMIKO‐1 in THP‐1m have yet to be elucidated in detail.

Our concept has been supported using a mouse model of interstitial pneumonia.^32^ Changes in the mRNA levels of proinflammatory cytokines in THP‐1m are a reliable marker for assessing the true function of hMIKO‐1 in cells. Despite the subsequent stimulation of THP‐1m preliminarily treated with hMIKO‐1 by LPS alone, the mRNA expression of these proinflammatory cytokines in THP‐1m was significantly suppressed (Figure 7). Therefore, the potential inhibition of inflammatory signal transduction in cells by hMIKO‐1 supports our concept. We are currently attempting to elucidate the underlying mechanisms. Alternatively, the slight induction of the mRNA expression of inflammatory cytokines by each hMIKO‐1, hS100A8, hS100A9, or calprotectin alone needs to be considered. Although the reason why hMIKO‐1 exhibits weak antigenicity to THP‐1m remains unclear, its three‐dimensional structure may be responsible for this antigenicity, or trace amounts of endotoxin may be contaminated in each material. Therefore, we corrected data by subtracting the volume of mRNA induced by each material alone from measured values to evaluate the true potential of hMIKO‐1. Although it is debatable whether this correction is reasonable, we considered it to be appropriate because each measured value was additive, not competitive with each other. Therefore, hMIKO‐1 is a functional molecule that comprehensively suppresses the excessive activation of THP‐1m by mainly blocking the nuclear factor‐kappa B pathway. We are currently investigating the true functions of hMIKO‐1.

Inflammatory signal transduction in THP‐1m is mainly delegated through surface protein molecules on the cell membrane in a complex manner. TLR4^5^, ^16^, ^17^, ^18^, ^19^ and RAGE^20^, ^21^, ^22^ are well‐known representative receptors on immune cells of myeloid origin, and CD68^33^, ^34^, ^35^ is one of the receptor‐like molecules involved in signal transduction in immune cells. To specify a target molecule of hMIKO‐1 on THP‐1m, an inhibition assay was tentatively performed using three types of monoclonal antibodies for TLR4, RAGE and CD68. After several trials, we found that only an anti‐CD68 antibody (KP1) significantly inhibited the binding of hMIKO‐1–FITC to THP‐1m (Figure 4c, f). These results strongly suggest that CD68 is an important receptor‐like molecule for hMIKO‐1. This concept is also supported by the results showing that the binding of hMIKO‐1–FITC to THP‐1m decreased after the treatment with some sialidases (Figure 5d–f). Therefore, we found that the sugar chains of CD68 were closely involved in immune reactions.

**Figure 8:**
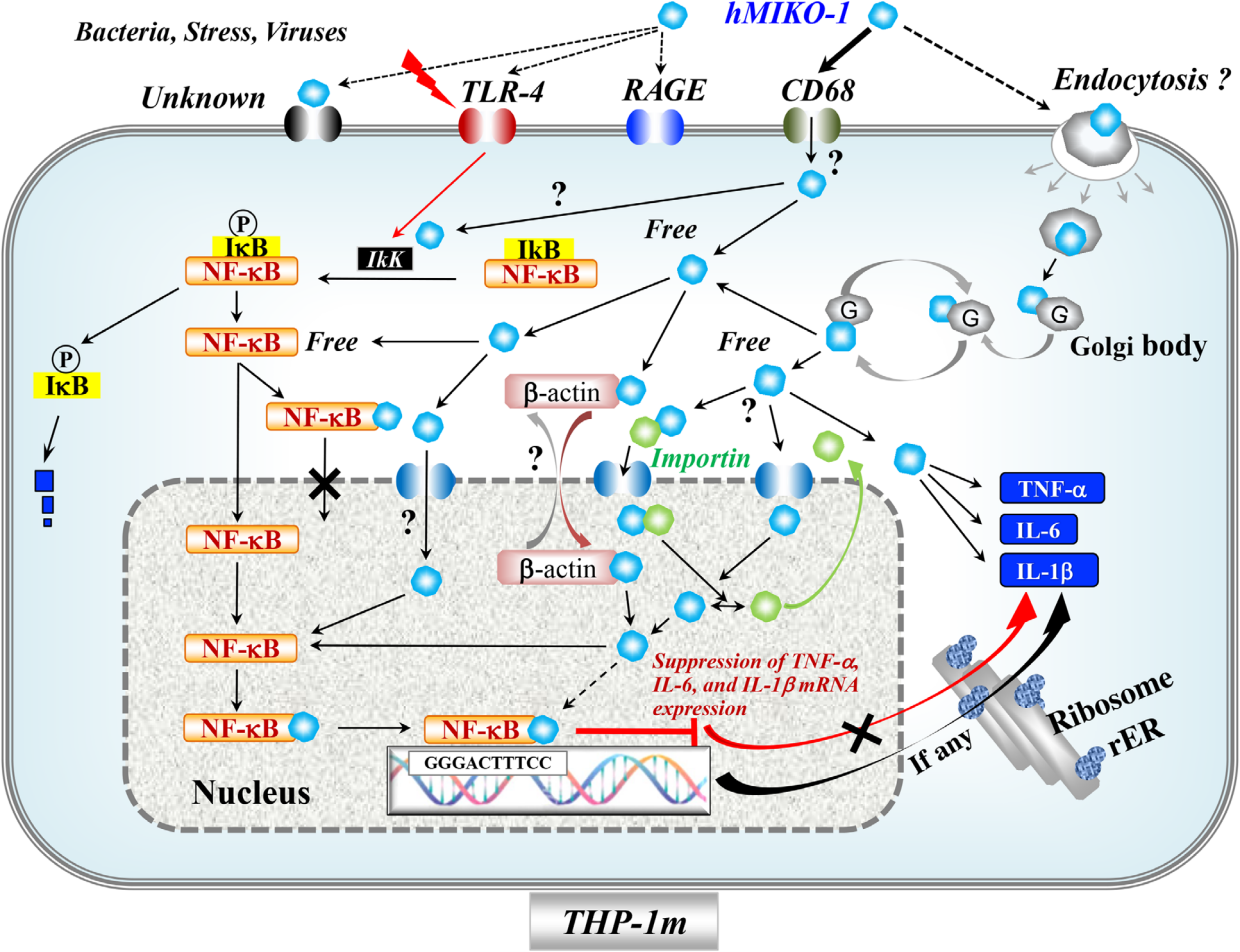
Schematic of a possible mechanism for the dynamic mobility of hMIKO‐1 in THP‐1m. ER, endoplasmic reticulum; hMIKO‐1, human MIKO‐1; IL, interleukin; mRNA, messenger RNA; NF‐κB, nuclear factor‐kappa B; RAGE, receptor for advanced glycation end products; TLR, Toll‐like receptor; TNF, tumor necrosis factor.

Although the role of hMIKO‐1 in THP‐1m has yet to be clarified, it may interact with the PDZ and LIM domain protein 1/2 (PDLIM 1/2) zone localized in the cytoplasm of THP‐1m or be intracellularly recycled in cells in a complex manner.^36^ The mechanisms underlying the intracellular metabolism of hMIKO‐1 warrant further study. We tentatively proposed the following mechanism for the dynamic mobility of hMIKO‐1 in THP‐1m (Figure 8). Unexpected infections by microorganisms or viruses may promote the activation of THP‐1m in the body, particularly in inflamed tissue. These events may result in severe pneumonia caused by the cytokine storm in lung tissue, where hMIKO‐1 may function as a novel suppressor to suppress excessively activated THP‐1m in the inflamed tissues of patients with inflammatory diseases that induce cytokine storms.

Many patients with inflammatory diseases, for example, severe pneumonia and/or respiration failure, may ultimately die without appropriate treatment. Although hMIKO‐1 is capable of suppressing cytokine storms, at least by inhibiting the mRNA expression of proinflammatory cytokines, the underlying mechanisms remain unclear. hMIKO‐1 has potential in the treatment of patients with various inflammatory diseases, such as connective tissue diseases, including interstitial pneumonia and inflammatory bowel diseases, as a novel therapeutic agent.

## CONCLUSION

hMIKO‐1 is a functional molecule that negatively regulates the excessive activation of THP‐1m by suppressing inflammatory signal transduction in cells. The greatest advantage of hMIKO‐1 is that the target cells are activated macrophages in inflamed tissues. Therefore, hMIKO‐1 may be clinically applied to the treatment of many inflammatory diseases. The present study will enable us to develop more effective medicines in the future. We are currently investigating the true functions of hMIKO‐1 in THP‐1m and the underlying mechanisms.

## METHODS

### Ethics approval and informed consent

All experiments were performed in accordance with the WMA Declaration of Helsinki‐Ethical Principles for Medical Research Involving Human Subjects (64th WMA General Assembly; Fortaleza, Brazil, October 2013). In the *in vitro* study, we used THP‐1 cells that were provided by the JCRB Cell Bank (National Institutes of Biomedical Innovation, Health and Nutrition, Osaka, Japan). As THP‐1 cells were isolated from a patient with acute monocytic leukemia, these cells are not recognized as human patients in the *in vitro* experiments. In addition, we cannot moralistically specify the origin in detail because of patient privacy.

### Reagents

THP‐1 (JCRB0112) cells were purchased from the JCRB Cell Bank, Ibaraki (Osaka, Japan). Difco LB Broth, Miller (Luria–Bertani) was obtained from Becton, Dickinson and Company, Sparks, MD, USA). Fetal bovine serum (FB‐1061/25), which was produced in the Dominican Republic, was obtained from Biosera Co., Ltd (Osaka, Japan). Ni‐agarose, Normocin (ant‐nr‐1; InvivoGen, San Diego, CA, USA) and Blocking One were obtained from Nacalai Tesque, Co., Ltd (Kyoto, Japan). PMA, Roswell Park Memorial Institute 1640 medium (RPMI‐1640; Biological Industries Ltd, Israel) and diaminobenzidine tetrahydrochloride tablets (DAB‐4HCl; 13 mg per tablet) were purchased from FUJIFILM Wako Pure Chemical Corporation (Osaka, Japan). The pCold‐1 vector and isopropyl β‐d‐1‐thiogalactopyranoside were purchased from Takara‐Bio Co., Ltd (Kyoto, Japan).^26^ Protein Deglycosylation Mix II (NEB‐P6044S) was purchased from New England BioLabs Inc., Ipswich, MA, USA. Accutase (AT104) was purchased from Funakoshi Co., Ltd (Tokyo, Japan). The Nuclear/Cytosol Fractionation Kit (catalog number 78833; NE‐PER Nuclear and Cytoplasmic Extraction Reagents) was obtained from Thermo Fisher Scientific Inc. (Rockford, IL, USA). LPS derived from *Salmonella* and the anti‐β‐actin antibody were purchased from Sigma‐Aldrich Co., LLC (Tokyo, Japan). *N*‐Hydroxysuccinimidobiotin (EZ‐Link^TM^ NHS‐Biotin) and FITC were obtained from Thermo Fisher Scientific Inc. (CA, USA). Streptavidin–TR, anti‐mouse IgG (horse) IgG, β‐actin antibodies, anti‐mouse IgG (horse) IgG–horseradish peroxidase, anti‐mouse IgG (horse) IgG–TR and VECTASHIELD mounting medium containing 4′,6‐diamidino‐2‐phenylindole dihydrochloride were obtained from Vector Inc. (Burlingame, CA, USA). The following monoclonal antibodies were used in the present study: (1) RAGE (A‐9: sc‐365154) purchased from Santa Cruz Biotechnology, Inc. (Dallas, TX, USA); (2) Human CD68 (KP1) obtained from Exalpha, Biologicals Inc. (MA, USA) and (3) TLR4 (NB100‐56566) from Novus Biologicals (USA). Monoclonal antibodies (clones: mAbC5, mAbC7, mAbC25 and mAbC27) for hMIKO‐1 were kindly provided by Mikuri Laboratory Co., Ltd (Osaka, Japan).

### Design of a hybrid protein (hMIKO‐1)

We designed a hybrid protein (hMIKO‐1) based on the AA sequences of hS100A8 and hS100A9, indicated by blue and gray horizontal bars, respectively (Figure 1a).^10^, ^24^ The whole frame of hMIKO‐1 is schematically indicated by a horizontal bar. hMIKO‐1 is a protein in which 21 AA residues from the C terminus of hS100A9 are added to the C terminus of hS100A8. The AA sequence of hMIKO‐1 was selected based on the AA sequences around the N and C terminals of hS100A8 and hS100A9, respectively, being immunologically active regions. Regarding AA sequences, we prepared hMIKO‐1 in consideration of the future possibility of a hybrid protein with specific functions being presumably difficult to evaluate if the AA sequences of both hS100A8 and hS100A9 are recombined at the same time. Therefore, we used the whole AA sequence of hS100A8 and a part of the AA sequence of hS100A9.

### Expression of hMIKO‐1 in *Escherichia coli* cells and its purification

The complementary DNA of hMIKO‐1 was artificially synthesized using gene technology and then inserted into the pCold I vector (Takara‐Bio Co., Ltd, Kyoto, Japan). The pCold I vector was separately transformed into competent cells (*Escherichia coli* cells, BL21) according to the manufacturer's instructions. Transformed *E. coli* cells were confirmed by cultivating at 37°C overnight on 1% agar gel containing ampicillin (1 × 10^−4^ g L^−1^) in a Petri dish. Genetically modified cells were frozen at −80°C until used. The expression of hMIKO‐1 in *E. coli* cells was performed as previously described.^14^ hMIKO‐1 was expressed in *E. coli* cells and then purified using Ni‐agarose [26 mm (D) × 100 mm (L)], DEAE cellulose [15 mm (D) × 150 mm (L)] and Sephacryl S‐300 HR [26 mm (D) × 1000 mm (L)] columns as previously described. After purification, the concentration of hMIKO‐1 was measured by a spectrophotometer (NanoDrop One; Thermo Scientific Co., Ltd, USA). Purified hMIKO‐1 (approximately 3 × 10^−3^ g L^−1^) was divided into microtubes (approximately 0.3 mL each) and then stored at −80°C until used. The concentration of endotoxin in the sample was measured using a commercially provided kit (Toxinsensor, Endotoxin Detection System; GenScript Inc.) according to the manufacturer's instructions.

### Differentiation of THP‐1 cells into THP‐1m by PMA and their confirmation

THP‐1 cells (1 × 10^6^ mL^−1^) were suspended in the culture medium (RPMI‐1640) containing 10% fetal bovine serum and normocin (1 × 10^‐4^ g L^−1^; medium A). PMA (100 nmol L^−1^) was added to the cell suspension as the final concentration. In FICS, 2 mL of the cell suspension was plated onto each well of the 6‐well culture plate. Cells in the plate were incubated at 37°C for 4–6 days in 5% CO_2_ to allow their differentiation to THP‐1m. Differentiated cells were subjected to FICS. In the flow cytometry experiments, 10 mL of the cell suspension was plated onto a Petri dish [100 mm (D) × 10 mm (H)] and cells were incubated as indicated above. Thereafter, an adequate volume of medium A (e.g. 1 mL) was added to the Petri dish, and cells were carefully collected using a scraper and transferred to a 1.5‐mL microtube. THP‐1m cells were confirmed by detecting CD68 using the anti‐CD68 IgG–FITC conjugate by flow cytometry. Alternatively, FICS was performed to confirm differentiated macrophages using the antibody (KP1).

### FICS

FICS was performed to microscopically observe the intracellular localization of hMIKO‐1 in THP‐1m as previously described.^14^ Briefly, adhered THP‐1m differentiated on a single glass plate (IWAKI Co., Ltd, Tokyo, Japan) by PMA for 5 days were treated using the following reagents, in which hMIKO‐1–FITC (approximately 5 × 10^−2^g L^−1^), hS100A8 (approximately 3.3 × 10^−2^ g L^−1^) and hS100A9 (approximately 5 × 10^−2^ g L^−1^)–biotin conjugates were used. Assay reagents were as follows: (a) an anti‐CD68 monoclonal antibody; (b) hMIKO‐1–FITC alone; (c) hMIKO‐1–FITC + hS100A8–biotin + hS100A9 and (d) FITC + hS100A8 + hS100A9–biotin. The mixtures (2 mL each) were added to each well of the plate followed by an incubation at 37°C for 12 h in 5% CO_2_. After washing two times with 50 mmol L^−1^ phosphate buffer (pH7.4)/0.9% NaCl solution sterilized (buffer A), adhered cells were fixed with 10% formalin at room temperature for 20 min. Cells were then treated with pure methanol at room temperature for 20 min and washed once with buffer A. To avoid nonspecific binding, cells were blocked for 20 min using goat serum preliminarily diluted 60‐fold with 50 mmol L^−1^ phosphate buffer (pH7.4) solution sterilized (buffer B). After washing, a mixture of anti‐mouse IgG (horse) IgG–FITC and streptavidin–TR conjugates (2 × 10^−3^ g L^−1^ each) was added to each well of the plate and incubated for 1 h to allow the immunochemical reaction. An anti‐mouse IgG (horse) IgG–TR conjugate was used to detect the anti‐CD68 monoclonal antibody bound to CD68 on THP‐1m. In addition, an anti‐mouse IgG (horse) IgG–streptavidin–TR conjugate as a control was used to clarify its nonspecific binding to cells. Stained cells were washed three times with buffer A and mounted with VECTASHIELD mounting medium. Fluorescent microscopic images were observed using a fluorescent microscope (BIOREVO BZ‐9000; KEYENCE Co., Ltd, Osaka).

### Extraction of cytoplasmic and nuclear proteins from THP‐1m

THP‐1m on three Petri dishes (*n* = 3) were washed three times with buffer A (approximately 10 mL). After washing, 1 mL of the same buffer solution was added to the Petri dish. Adhered cells were carefully released from the dishes using Accutase (10 mL) followed by a scraper and were then collected into a 50‐mL conical tube. Residual cells were centrifuged at 905*g* at 4°C for 3 min, and the supernatant was discarded. Collected cells were resuspended in phosphate‐buffered saline (1 mL) and finally transferred into a 1.5‐mL microtube. The cell suspension was carefully centrifuged at 905*g* at 4°C for 3 min, and the supernatant was discarded. Cytoplasmic and nuclear proteins in THP‐1m cells were carefully extracted using a Nucleus/Cytosolic Fractionation Kit according to the manufacturer's instructions (catalog number 78833; NE‐PER Nuclear and Cytoplasmic Extraction Reagents, Rockford, IL, USA). Extracted protein samples were frozen at −80°C until used.

### SDS–PAGE and Western blotting

SDS–PAGE was performed to visualize proteins extracted from THP‐1m in the presence of 2‐mercaptoethanol, as previously described.^26^ Western blotting was also performed to confirm target proteins using protein samples extracted from the cytoplasm and nucleus of THP‐1m.

### Identification of unknown proteins bound to hMIKO‐1

#### Immunoprecipitation

To confirm possible complexes between hMIKO‐1 and β‐actin or other proteins, immunoprecipitation was conducted using two monoclonal antibodies (mAbC25 and mAbC26) specific for hMIKO‐1 that recognize different epitopes of hMIKO‐1. Briefly, the two antibodies (approximately 5 × 10^−3^g L^−1^ each) were added together to cytoplasmic and nuclear protein samples (0.5 mL each) extracted from the cytoplasm and nucleus, respectively, of THP‐1m treated with hMIKO‐1–biotin at 37°C for 4 h in 5% CO_2_. The mixtures were gently mixed at 4°C overnight and then centrifuged at 1500 rpm at 4°C for 5 min. The resultant precipitate (pellet) was carefully washed two times with buffer A. After centrifugation as described above, precipitated proteins were subjected to SDS–PAGE in the presence of 2‐mercaptoethanol. Proteins in the gel were electrically transferred to a nitrocellulose membrane. Western blotting was performed to detect hMIKO‐1–biotin on the membrane using streptavidin–horseradish peroxidase, and β‐actin using an anti‐β‐actin monoclonal antibody and anti‐mouse IgG (horse)–horseradish peroxidase conjugate as the first and second antibodies, respectively. Antibody‐bound proteins were detected by assessing horseradish peroxidase activity using *o*‐PD and hydrogen peroxide as substrates for color development.^23^

### Polymerase chain reaction and quantitative polymerase chain reaction

Semiquantitative polymerase chain reaction (PCR) and quantitative PCR were performed using a Thermal Cycler (C1000; BIO‐RAD) as previously described.^6^, ^14^ Briefly, total RNA was extracted from THP‐1m treated with or without hMIKO‐1 using an RNeasy Mini Kit (Qiagen Ltd, Manchester, UK). Complementary DNA for each target protein was synthesized using an ExScript RT kit (Takara, Shiga, Japan) according to the manufacturer's instructions. Quantitative PCR was performed using an ABI PRISM 7000 Sequence Detection System (Applied Biosystems, CA, USA). The quantitative PCR primers used are indicated in Supplementary table 1. The relative mRNA expression level of each target gene was calculated using the comparative C_T_ method. The measurement of target mRNA was independently repeated in triplicate for each sample.

### Flow cytometry

#### Sialidase treatment

##### Suspension mode

THP‐1m cells (5 × 10^5^ mL^−1^) were treated with a sialidase cocktail (Protein Deglycosylation Mix II: NEB‐P6044S) according to the manufacturer's instructions. Briefly, 0.4 mL of distilled water was added to a cell pellet. Cells were then gently suspended at approximately 5 × 10^5^ mL^−1^, and 10 μL of buffer solution I (×10) was quickly added to the cell suspension followed by gentle mixing. The sialidase cocktail (10 μL) was added to the suspension followed by an incubation at room temperature for 30 min. After the treatment, the suspension was centrifuged at 905*g* at 4°C for 3 min, and the supernatant was discarded. Residual cells were resuspended at approximately 5 × 10^5^ mL^−1^ in an adequate volume of medium A. Assay mixtures were as follows: A (hMIKO‐1–FITC alone) and B (hMIKO‐1–FITC + hS100A8 + hS100A9); C (hMIKO‐1–FITC + calprotectin). All mixtures were preliminarily treated using a 0.45‐μm filter unit for sterilization. Suspended cells in each mixture were incubated at 37°C for 3 h in 5% CO_2_ with mixing every 30 min. All cells were then washed two times with buffer B and subjected to flow cytometry using a flow cytometer (BD FACS Aria Fusion Flow Cytometers; Becton Dickinson and Co., BD Biosciences San Jose, CA, USA).^23^ The concentrations of hMIKO‐1–FxITC, hS100A8, hS100A9 and calprotectin were approximately 5, 3.3, 5 and 5 × 10^−2^ μg mL^−1^, respectively.

##### Dish mode

THP‐1m (5 × 10^5^ mL^−1^) on the 6‐well plate were treated with each assay mixture as described above. Cells in each well of the plate were then washed two times with buffer B. Adhered cells in each well were carefully collected by a scraper and transferred to each small 5‐mL tube. Collected cells were subjected to flow cytometry using a flow cytometer (BD FACS Aria Fusion Flow Cytometers; Becton Dickinson and Co., BD Biosciences San Jose, CA, USA).^23^

### Statistical analysis

Pairwise comparisons with the controls were performed using parametric tests. Significant differences between groups were identified using the Student *t*‐test (a *t*‐test of the difference between two mean values). Data are shown as the mean ± SD. Significance is indicated as *P < 0.05, **P < 0.01 and ***P < 0.001.

## AUTHOR CONTRIBUTIONS

MI, TK, KO, SM, and TT together conceived the study, participated in its design and coordination, and further made a draft of the manuscript. MI, TK, and KO were responsible for the genetic analysis. MI participated in the preparation of hMIKO‐1, hS100A89, hS100A9, and calprotectin. MI was responsible for inducing the differentiation THP‐1 cells to human macrophage‐like cells using PMA. All researchers critically discussed our data.

## CONFLICT OF INTEREST

The authors declare no competing interests.

## Supporting information


Supplementary figure 1.
**Supplementary table 1**.

## Data Availability

Data and materials generated during the present study are available from the corresponding author upon reasonable request.
