# Supplementary material for: A hybrid protein is a functional molecule to reduce the cytokine storm caused by excessively activated macrophages
Source: Immunol Cell Biol. 2025 Feb 15;103(4):350–64. doi: 10.1111/imcb.70000 (PMC11964790; doi:10.1111/imcb.70000)
Supplement: Supplementary file 1 — Supplementary figure 1. Supplementary table 1. [file IMCB-103-350-s001.pptx]

## Slide 1
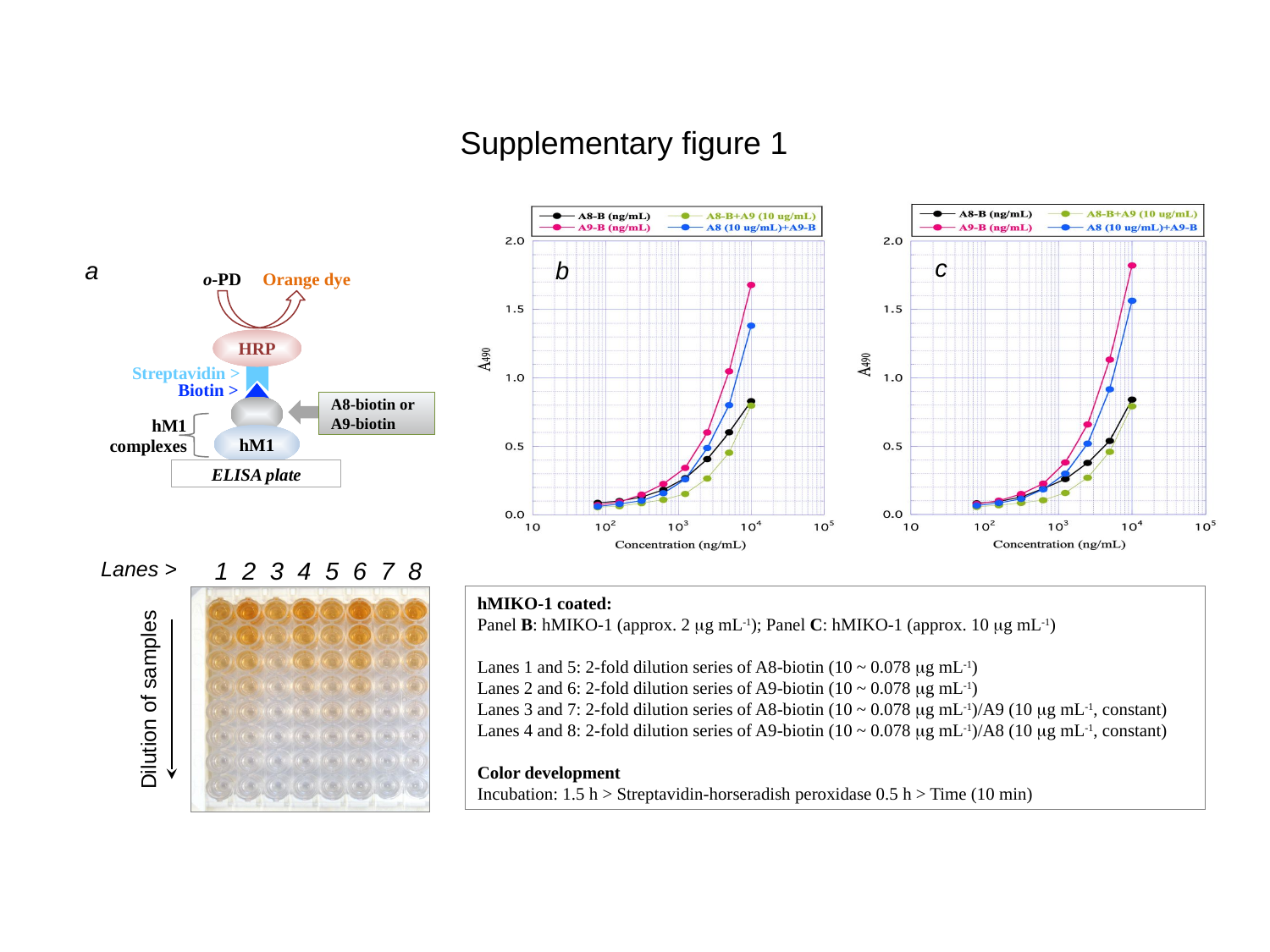

Supplementary figure 1
b
c
a
Orange dye
o-PD
HRP
Streptavidin >
Biotin >
A8-biotin or A9-biotin
hM1
complexes
hM1
ELISA plate
Lanes >
1 2 3 4 5 6 7 8
hMIKO-1 coated:
Panel B: hMIKO-1 (approx. 2 mg mL-1); Panel C: hMIKO-1 (approx. 10 mg mL-1)
Lanes 1 and 5: 2-fold dilution series of A8-biotin (10 ~ 0.078 mg mL-1)
Lanes 2 and 6: 2-fold dilution series of A9-biotin (10 ~ 0.078 mg mL-1)
Lanes 3 and 7: 2-fold dilution series of A8-biotin (10 ~ 0.078 mg mL-1)/A9 (10 mg mL-1, constant)
Lanes 4 and 8: 2-fold dilution series of A9-biotin (10 ~ 0.078 mg mL-1)/A8 (10 mg mL-1, constant)
Color development
Incubation: 1.5 h > Streptavidin-horseradish peroxidase 0.5 h > Time (10 min)
Dilution of samples

## Slide 2
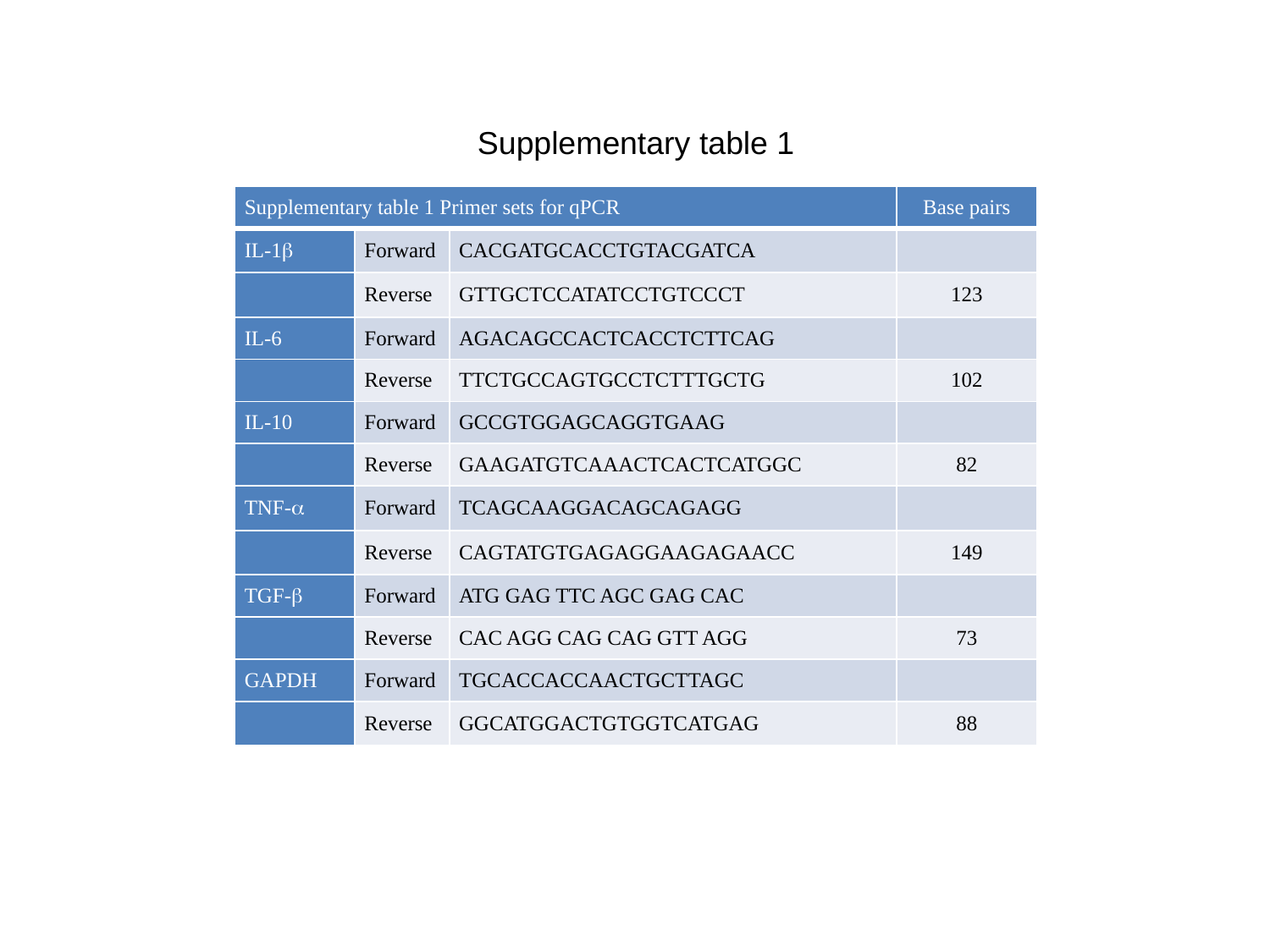

Supplementary table 1
| Supplementary table 1 Primer sets for qPCR | | | Base pairs |
| --- | --- | --- | --- |
| IL-1b | Forward | CACGATGCACCTGTACGATCA | |
| | Reverse | GTTGCTCCATATCCTGTCCCT | 123 |
| IL-6 | Forward | AGACAGCCACTCACCTCTTCAG | |
| | Reverse | TTCTGCCAGTGCCTCTTTGCTG | 102 |
| IL-10 | Forward | GCCGTGGAGCAGGTGAAG | |
| | Reverse | GAAGATGTCAAACTCACTCATGGC | 82 |
| TNF-a | Forward | TCAGCAAGGACAGCAGAGG | |
| | Reverse | CAGTATGTGAGAGGAAGAGAACC | 149 |
| TGF-b | Forward | ATG GAG TTC AGC GAG CAC | |
| | Reverse | CAC AGG CAG CAG GTT AGG | 73 |
| GAPDH | Forward | TGCACCACCAACTGCTTAGC | |
| | Reverse | GGCATGGACTGTGGTCATGAG | 88 |
